# Understanding implementation processes of clinical pathways and clinical practice guidelines in pediatric contexts: a study protocol

**DOI:** 10.1186/1748-5908-6-133

**Published:** 2011-12-28

**Authors:** Shannon D Scott, Jeremy Grimshaw, Terry P Klassen, Alberto Nettel-Aguirre, David W Johnson

**Affiliations:** 1 Faculty of Nursing, University of Alberta, Edmonton, Canada; 2 Department of Pediatrics, Faculty of Medicine and Dentistry, University of Alberta, Edmonton, Canada; 3 Ottawa Health Research Institute, Faculty of Medicine, University of Ottawa, Ottawa, Canada; 4 Department of Pediatrics and Child Health, Faculty of Medicine, University of Manitoba, Winnipeg, Canada; 5 Department of Pediatrics and Community Health Sciences, Faculty of Medicine, University of Calgary, Calgary, Canada; 6 Department of Pediatrics, Faculty of Medicine, University of Calgary, Calgary, Canada

## Abstract

**Background:**

Canada is among the most prosperous nations in the world, yet the health and wellness outcomes of Canadian children are surprisingly poor. There is some evidence to suggest that these poor health outcomes are partly due to clinical practice variation, which can stem from failure to apply the best available research evidence in clinical practice, otherwise known as knowledge translation (KT). Surprisingly, clinical practice variation, even for common acute paediatric conditions, is pervasive. Clinical practice variation results in unnecessary medical treatments, increased suffering, and increased healthcare costs. This study focuses on improving health outcomes for common paediatric acute health concerns by evaluating strategies that improve KT and reduce clinical practice variation.

**Design/Methods:**

Using a multiple case study design, qualitative and quantitative data will be collected from four emergency departments in western Canada. Data sources will include: pre- and post-implementation focus group data from multidisciplinary healthcare professionals; individual interviews with the local champions, KT intervention providers, and unit/site leaders/managers; Alberta Context Tool (ACT) survey data; and aggregated patient outcome data. Qualitative and quantitative data will be systematically triangulated, and matrices will be built to do cross-case comparison. Explanations will be built about the success or lack of success of the clinical practice guidelines (CPG) and clinical pathways (CPs) uptake based upon the cross-case comparisons.

**Significance:**

This study will generate new knowledge about the potential causal mechanisms and factors which shape implementation. Future studies will track the impact of the CPG/CPs implementation on children's health outcome, and healthcare costs.

## Background

Canada is among the most prosperous nations in the world, yet when compared to other nations in the Organisation for Economic Co-operation and Development (OECD), Canadian children's health and wellness outcomes are surprisingly poor [1]. Each year almost 25% of Canada's approximate 9,500,000 million children require emergency acute healthcare resulting in considerable financial and emotional costs for families and society. Clinical practice variation, even for common acute child health conditions, continues to be pervasive, despite guidance from research evidence [2]. Poorer health outcomes, unnecessary medical treatments and suffering, and increased strain on the healthcare system are potential outcomes from practice variation. Strategies that mobilize the use of research evidence to inform children's healthcare can reduce healthcare utilization [3-11] and high hospitalization rates. Hospitalization accounts for 43% to 62% of healthcare expenditures [12-16] and comes with inherent risks [17-19]. Reduction of undesirable practice variation has been a major focus of systematic efforts to improve the quality of the healthcare system [20]. Clinical practice guidelines (CPGs) and clinical pathways (CPs) have been embraced as one strategy to decrease clinical practice variation through providing the best available research evidence in the form of optimal care recommendations. However, current research highlights that strategies to put CPG/CPs in clinical practice have not resulted in uniform implementation rates across sites.

Despite the billions annually spent globally and the hundreds of millions spent in Canada [21] on high-quality health research, research transfer is a slow and haphazard process [22]. In fact, it often takes 10 to 20 years for research findings to be 'translated' into conventional healthcare delivery [23]. Regrettably, child healthcare settings are not immune to the challenges of applying the best available research evidence to clinical practice, also known as knowledge translation (KT). The literature highlights that the effectiveness of KT interventions, such as CPG/CPs, to facilitate the transfer of research into clinical practice varies by condition, professional group, and context; however, the processes and factors shaping the implementation processes are not well studied.

CPGs are systematically developed research-based recommendations to assist health professional and patient decisions about appropriate healthcare for specific clinical circumstances [24,25]. While broadly similar to CPGs, CPs differ by being more explicit about the sequence, timing, and provision of interventions [26]. Pathways are usually based on pre-existing CPGs and developed by a multidisciplinary team of healthcare providers from one health institution for use within that institution. Despite the prevalence of CPs, few published studies have evaluated their impact on professional practice patterns or clinical outcomes [26].

While CPG/CPs are well-established tools to ensure that the best available research evidence informs healthcare, purposeful KT interventions, such as educational sessions and reminders, are required to implement or ensure that the CPG/CPs get used. Studies that determine whether KT interventions (*e.g*., printed reminders, interactive educational sessions, and local champions) result in improved patient outcomes have been conducted, but little has been published about the causal mechanisms that facilitated CPG/CP use (the 'how') or the effect modifiers (factors) that shaped the process. This would not be an issue if we expected uniform CPG/CP implementation rates across different settings and conditions that could then be generalized to practitioners outside of a study area. However, current evidence suggests variation in CPG/CPs implementation rates across the study sites and by condition, professional group, and context [27,28] seemingly occur because the causal mechanisms of the interventions are modified in the presence of different barriers and facilitators (the effect modifiers). Thus, studying the causal mechanisms and effect modifiers at each study site is an imperative step to understand how to design future CPGs/CP implementations with a more consistent effect [29] that can be customized to the attributes of the CPG/CP condition, professional group, and context [30].

There is increasing recognition of the importance of process evaluation studies alongside trials of complex interventions, such as KT interventions that demand behavior change [27,31] in complex environments [32,33]. These studies can: delineate the extent to which all intervention components are implemented; outline the factors that shaped the implementation process; assess the consistency of intervention delivery across multiple sites; explain positive, modest, and insignificant results' and provide important links to understand and improve interventions. Process evaluation studies are comparable to measuring intermediate endpoints in clinical trials to further understand the biological basis of any observed effect [27]. The published literature includes a wealth of studies about KT interventions that have successful outcomes [28,34], however there are a limited number of studies that disentangle the factors that facilitate or hinder successful outcomes, characterize the failure to achieve success, or attempt to document the steps involved in achieving successful CPG/CP implementation [33]. To date, much of the KT research has not explored the 'black box'--that is, KT interventions (inputs) are tested and outcomes (outputs) are measured, however there has been no examination of the mediating factors and processes (in the box) that lead to the outcomes.

A unique opportunity exists to develop new knowledge to improve children's health outcomes, as well as make theoretical contributions to KT. A CP for paediatric gastroenteritis and a CPG for croup will be implemented in 15 emergency departments (EDs) and Urgent Care Centres (UCC) in western Canada. This mix of health settings reflects the variation of settings in which children receive healthcare. Each of the 15 sites will have a gastroenteritis CP and a croup CPG implemented using evidence-based KT strategies including printed materials, educational sessions and champions [35-37]. The study proposed here evaluates the implementation of the CPG/CP by examining the factors that shape the implementation process to develop theory to explain the well established variation in CPG/CP implementation.

### Research questions

With this study, we intend to answer the following research questions:

1. What are the causal mechanisms and effect modifiers (factors) that shape the CPG/CP implementation processes?

2. How do the causal mechanisms and effect modifiers of the KT interventions shape CPG/CP implementation?

### Specific objectives

Our objectives are:

1a. to assess dose received of each of the KT interventions at each site and the differences in dose received using qualitative and quantitative data from health professionals, managers, KT intervention providers, and local champions.

1b. to assess dose delivered, reach, and fidelity of each the KT interventions.

1c. to assess the attributes of the CPG/CP, factors from each context (effect modifiers) and extent (both of effort and uptake) of CPG/CP implementation at each site.

2. to develop an explanatory theory of the effect modifiers and causal mechanisms shaping the CPG/CP implementation in healthcare settings delivering care to children using 'pattern matching' [38,39] techniques across the study variables within four of the 15 study sites.

### Theoretical framing

The Ottawa Model of Research Use [40] will be used to frame the data collection and analysis for this study. This model, derived from theories of change, is a comprehensive, interdisciplinary framework of elements that affect KT. The six elements in the model considered central to KT are: the evidence-base of the innovation (CPG/CP); potential adopters (those using the CPG/CP); practice environment (context); interventions to transfer the innovation (*i.e*., KT strategies); adoption of the innovation (use of the CPG/CP); and outcomes (*i.e*. CPG/CP implementation rates, actual health outcomes).

## Methods/design

A mixed method multiple case study research design [39,41] will be used to systematically explore the simultaneous implementation of two research-based CPG/CPs in four clinical sites. A 'case' for each study site will be developed. Case studies provide the needed depth and complexity to develop theory to explain how the attributes of the KT interventions interact with contextual elements (effect modifiers) to 'produce' CPG/CP implementation processes that shape patient and system outcomes. The components to be assessed in the study are included in Table 1.

**Table 1:** Components to be assessed in the study

Component	Definition	Data collection	Study objective
**Context**	Environment/setting where CPG/CP is occurring	-ACT survey -Pre/Post focus group data	1c

**CPG/CP attributes**	Features of the CPG and CP	-Post focus group data	1c

**Reach (of KT interventions)**	Proportion of professionals that participates in each KT intervention.	-Education session attendance -Local champion interviews	1b

**Dose delivered (of KT interventions)**	Amount of intervention delivered	-Intervention provider interviews	1b

**Dose received (of KT interventions)**	Extent participants engaged with the KT interventions	-Post-focus group data -KT intervention records	1a

**Fidelity (of the KT interventions)**	Extent to which the interventions were delivered as planned	-Intervention provider interviews -KT intervention records	1b

**Implementation of the CPG/CP**	Extent to which the intervention has been implemented and received	-CPG/CP use survey scores -Outcome data on CPG/CP use	1c

### Sampling

#### Settings and site selection

There are 15 sites implementing the CPG/CP; four implementation sites will be purposefully selected to reflect both urban and rural healthcare centres. All health professionals in each study site will be invited to participate in the focus group interviews and surveys.

#### Data types and collection

The following data types will be collected for each case (study site): pre-and post-implementation focus group data with representation from all relevant health professional groups (*e.g*. nurse, physicians, and allied health professionals working in the ED); individual interviews with the local champions, intervention providers and managers of each study site; Alberta Context Tool (ACT) [42] survey data from health professionals at each study sites; survey data on the extent of CPG/CP use and KT intervention effectiveness; and documentation on KT intervention delivery. Furthermore aggregate patient and system outcome data (*e.g*., hospitalization rates, length of stay) will be used as additional data sources.

#### Data collection

##### Group and individual interviews

Group interviews (focus groups) are an efficient, cost-effective data collection method that provide opportunities to generate rich data while also observing group dynamics and levels of consensus on topics. Focus groups will include four to six participants and will range from 60 to 90 minutes. Depending on the study site, participants could include a paediatrician and emergency physician, a nurse from the ED and inpatient setting, a pharmacist, a nurse manager, and a nurse educator. Two focus groups will be conducted at each site both pre-implementation and post-implementation (16 focus groups) to acknowledge the power dynamics amongst the health professional groups. Key informant selection will be done in collaboration with the local champion at each site. Interview questions will focus on: attributes of and experiences using the CPG/CP; attributes of their work environment (context); barriers to using the CPG/CP; perceptions of CPG/CP implementation success; and perceptions of the utility of the KT interventions.

The focus groups will be conducted by SDS, and the project coordinator will record observations of the focus group as field notes (descriptive written accounts of events). A court reporter will be present at all focus groups to do 'real-time transcription.' This method produces transcripts of greater fidelity more rapidly, which prevents the loss of important contextual information and provides a more complete and accurate account of the proceedings on which to base subsequent analysis [43]. Individual interviews will also occur with: local champions at each site (n = 4); the KT intervention providers (n = 3); and clinical setting managers post-implementation (n = 4).

##### Survey data

All health professionals working at the four study sites will be invited to complete an on-line version of the ACT one month following the initial delivery of the KT interventions. The ACT measures organizational context and research utilization behaviours through assessing eight dimensions of context, including: culture, leadership, evaluation, social capital, informal interactions, formal interactions, structural and electronic resources, and organizational slack. This tool has been used with nurses, physicians, clinical specialists, allied health professionals, and managers, and the dimensions have internal reliability, ranging from 0.5 to 0.96 [44,45]. The survey takes approximately 20 minutes to complete and it has been revised, pilot tested, and used with both adult and paediatric health professionals in multiple Canadian healthcare settings [46]. Given the size of each of these sites, and previous response rates from other studies we have conducted at these sites, we anticipate approximately 30 completed surveys/site. In addition, health professionals will be asked to complete 10 survey questions to assess the attributes of the KT interventions and extent of CPG/CP implementation.

##### KT intervention documentation

The providers of the KT interventions will be asked to complete a spreadsheet to assess delivery and participation in the KT interventions.

##### Data analysis

Analysis of the multiple data sources will require a combination of qualitative and quantitative approaches.

##### Qualitative data analysis

The 16 focus group and 11 individual interviews will yield a large quantity of complex data. SDS will lead the qualitative (inductive) analysis working with the project co-ordinator and a graduate student. To monitor the progress of the interviews and permit follow-up of ideas that emerge from the data, data collection and analysis will proceed concurrently. The inductive analysis will occur in three phases: coding, categorizing, and developing themes. First, all data will be coded to facilitate analysis. The code word(s) will reflect the essence of the data, leading to ease of recognition as the number of code words increases. Codes will be operationally defined so that they can be consistently applied throughout the data. Second, codes will be placed into broad categories that correspond to the major unit of analysis. As categories emerge, their theoretical properties will be defined. Comparisons between multiple categories will be carried out in order to locate similarities and differences between them. Finally, to obtain a holistic view of the data, categories will be synthesized into themes. This process will be replicated for the qualitative data for each 'case.' Data analysis will be managed using the NVIVO software package.

##### Survey analysis

All ACT data will be entered into Statistical Package for Social Sciences (SPSS) version 18.0. These data collected at the individual level will be aggregated to the level of the clinical setting (case) by calculating group means. Descriptive statistics (frequencies) and proportions will be first conducted. Parametric (ANOVA) and nonparametric (Kruskal-Wallis test) will be used to provide a robust comparison among the four cases (study sites) in terms of each of the eight dimensions of context. Quantitative data will be graphically displayed to reflect the variation in the eight dimensions across the four sites. Statistical results will be one evidence source for each case.

##### Data triangulation

Data triangulation will occur through an integrated approach where the qualitative and quantitative data will be systematically juxtaposed by case (study site) through matrices [38] to examine patterns and differences (across-cases) in terms of delivery of the KT intervention (*e.g*., reach, dose delivered, and fidelity), effect modifiers such as context, and outcomes (health professional survey scores on CPG/CP implementation, case aggregated patient outcomes). Next, detailed case descriptions will be written on each of the four cases (study sites). Throughout data triangulation, 'pattern matching' will occur across the four sites' data in order to build theory explaining the CPG/CP implementation process. The complementary use of qualitative and quantitative data lend to the development of rich, detailed and credible findings [38,39].

##### Strategies to ensure study rigor

To minimize potential bias, the collection and initial coding and analysis of the qualitative data will be supervised and conducted by SDS, who is not implementing the CPG/CPs. The investigators who were involved in the CPG/CP implementation will be incorporated into the final stages of analysis as debriefers to provide credibility to the case descriptions. All methodological decisions and insights will be documented in an audit trail [47,48]. Throughout analysis, memos (detailed written accounts of events and decisions) will be written to document the analysis process and facilitate the development of explanatory theory. Coding will be done jointly by the qualitative analysis team and consensus negotiated to ensure representativeness [47].

##### Ethics

Ethical approval has been granted for this study. Informed consent procedures will include seeking the participants' consent to be interviewed (interviews and focus groups) and having the interview and/or focus group audio taped and/or transcribed in 'real time.' All identifying information from the interview and focus group transcripts will be removed prior to data analysis. The court reporter (focus groups) will also sign a confidentiality agreement. All research data will be stored under double lock and key for a period of seven years.

#### Knowledge translation

##### Decision-maker partnership and integrated KT

To ensure effective communication and involvement between the research team and potential users and to accelerate the capture of benefits from this study, a strategic partnership a multidisciplinary group of paediatric clinicians, healthcare decision makers, and researchers from across the province who are charged with optimizing child health outcomes in Alberta has been developed. This will serve as the ideal environment to provide input about study findings and to facilitate evidence-informed policy making to improve children's health outcomes.

## Discussion

Systematically studying CPG/CP implementation processes is integral to improving future child health and system outcomes. This study will generate new knowledge about the causal mechanisms and factors that shape the implementation of CPG/CPs in health settings delivering care to children. Further, this study will generate new knowledge on how economies-of-scale can be leveraged through innovative sharing of KT interventions to implement two CPG/CPs. This knowledge can then be leveraged to inform and improve future CPG/CP implementation efforts. Most importantly, in order for CPG/CP to be an effective approach to put the best research evidence into clinical practice, it is essential that CPG/CP implementation processes are systematically studied to develop theory to explain implementation variation across sites. The application of research findings in healthcare interventions, services, and policy decisions is a strategic value and a fundamental principle of many provincial and national healthcare agencies, as well as an important 'return-on-investment' societal priority. The findings developed through this research will be especially important for health organizations because great priority has been placed on the development and standardization of CPs to facilitate equitable, seamless, and consistent access to healthcare. Knowledge of the processes and factors shaping CPG/CP implementation will provide needed guidance to assist health system bodies as future research-based pathways are developed and implemented.

## Competing interests

The authors declare that they have no competing interests.

## Authors' contributions

SDS conceived this evaluation study, provided leadership and coordination in the design and conduct of the study, obtained funding, drafted and edited the final manuscript, and approved the final submitted manuscript. DJ contributed to study conception, and TK, JG, and ANA participated in critically appraising and revising the intellectual content of the manuscript. All authors read and approved the final manuscript.
